# Effects of a 6-Min Treadmill Walking Test on Dual-Task Gait Performance and Prefrontal Hemodynamics in People With Multiple Sclerosis

**DOI:** 10.3389/fneur.2022.822952

**Published:** 2022-04-07

**Authors:** Kim-Charline Broscheid, Martin Behrens, Christian Dettmers, Michael Jöbges, Lutz Schega

**Affiliations:** ^1^Department of Sport Science, Chair of Health and Physical Activity, Institute III, Otto von Guericke University Magdeburg, Magdeburg, Germany; ^2^Department of Orthopedics, Rostock University Medical Center, Rostock, Germany; ^3^Kliniken Schmieder Konstanz, Konstanz, Germany

**Keywords:** fNIRS, functional near-infrared spectroscopy, fatigue, fatigability, 6MWT, MS

## Abstract

Fatigue is one of the most limiting symptoms in people with multiple sclerosis (pwMS) and can be subdivided into trait and state fatigue. Activity-induced state fatigue describes the temporary decline in motor and/or cognitive performance (motor and cognitive performance fatigability, respectively) and/or the increase in the perception of fatigue (perceived fatigability) in response to motor or cognitive tasks. To the best of our knowledge, the effects of a 6-min walk test (6MWT), which was often used to assess motor performance fatigability in pwMS, on motor-cognitive dual-task performance (i.e., walking + arithmetic task) and prefrontal cortex (PFC) hemodynamics are not well-known. This is of importance, since daily activities are often performed as multitasks and a worse dual-task walking performance is associated with an increased risk of falling. Consequently, we investigated the effect of a fast 6MWT (comfort velocity + 15%) performed on a treadmill on motor-cognitive performance fatigability (spatio-temporal gait parameters/accuracy during the arithmetic task) and perceived fatigability measures (rating of perceived exhaustion; RPE) as well as PFC hemodynamics recorded during dual-task walking in pwMS and healthy controls (HCs). Twenty pwMS (48.3 ± 9.0 years; 13 females/7 males; expanded disability status scale 2.7 ± 1.0, first diagnosis 13.8 ± 8.8 years) and 24 HC with similar age and sex (48.6 ± 7.9 years; 17 females/7 males) were included. Only cognitive performance fatigability (increased error rate) during dual-task walking was found after the fast 6MWT on the treadmill in pwMS. However, the changes in gait parameters did not indicate motor performance fatigability, although both the groups reported perceived fatigability (increased RPE) after the fast 6MWT. Moreover, no change in the PFC activation was detected in both groups. Our results suggest that the intensity and/or duration of the fast 6MWT was not sufficient to induce motor performance fatigability in pwMS. These factors should be addressed by future studies on this topic, which should also consider further parameters, e.g., muscular oxygenation and/or myoelectrical activity, to verify that exercise intensity and/or duration was appropriate to induce motor performance fatigability in pwMS.

**Clinical Trial Register:**

DRKS00021057.

## Introduction

Over 75% of people with multiple sclerosis (pwMS) report that fatigue is the most limiting symptom with a high negative impact on daily life (1). In the MS context, fatigue is often defined as a subjective lack of physical and/or mental energy that is perceived by the affected person or caregiver interfering with usual and desired activities (2). However, this definition does not cover the different dimensions of fatigue comprising perceptual and performance aspects that were investigated separately in the past (3–6). To resolve this, Enoka and Duchateau (3) provided a fatigue definition and framework, which were recently adapted to describe the dimensions and mechanisms contributing to fatigue in pwMS (7). Within this framework, a distinction is made between trait and state fatigue. Trait fatigue describes the fatigue perception of pwMS over a longer period of time (e.g., weeks or months) and is associated with primary disease-related and secondary mechanisms (e.g., depression and medication). In contrast, activity-induced state fatigue describes the temporary decline in motor and/or cognitive performance (performance fatigability) and/or the increase in the perception of fatigue (perceived fatigability) in response to a motor or cognitive task. Thereby, motor performance fatigability is determined by the activation characteristics as well as the contractile function of muscles (3) and cognitive performance fatigability by the integrity of the central nervous system (e.g., neural excitability, metabolites, and neurotransmitter) (6, 8). Perceived fatigability strongly depends on the psychophysiological state of the individual (9). Both the performance fatigability and perceived fatigability are interdependent and should be investigated in conjunction (7).

The majority of studies assessing motor performance fatigability in pwMS used single muscle or muscle group performance tests, while only a few studies employed whole-body exercises such as walking (10). The latter is of particular importance for activities of daily life. In this context, the 6-min walk test (6MWT) was mostly applied as a fatigue intervention and/or assessment with discrepant effects on motor performance fatigability indices in pwMS, i.e., some showed a decline in walking velocity (11, 12) and others not (13) depending on the degree of disability (14). However, these studies only investigated performance fatigability while executing a single-task 6MWT. Therefore, to the best of our knowledge, the effects of a 6MWT on motor-cognitive dual-task performance (e.g., walking + arithmetic task) are not well-known. This is of particular importance, since daily activities are often performed as multitasks and a worse dual-task walking performance is associated with an increased risk of falling (15). In general, pwMS display a decreased gait performance during dual-task walking compared to single-task walking, with gait performance being worse than that of healthy controls (HC) in both conditions (16). This motor-cognitive interference during dual-task walking was explained by impaired cognitive functions (17), i.e., especially the attentional capacity [located among others in the prefrontal cortex (PFC)] in pwMS (18). For instance, Hernandez et al. have demonstrated that the PFC activation during single- and dual-task overground walking was higher in pwMS than in HC (19), which may be due to structural and functional changes related to MS (20). Moreover, they have shown that PFC activation was higher during dual-task walking compared to single-task walking in both groups presumably due to higher attentional demands (19).

Nevertheless, it is currently not known if motor performance fatigability induces a reallocation of attentional resources and/or compensatory processes during dual-task walking in pwMS compared to HC. For instance, the findings of Vuillerme et al. point in this direction showing that motor performance fatigability of the calf muscles resulted in a decreased cognitive performance (auditive reaction time task), while conducting a motor task (maintaining static postural control) in healthy young adults (21).

Based on the literature presented above, we investigated the effect of a fast 6MWT performed on a treadmill on performance and perceived fatigability measures as well as PFC hemodynamics recorded during dual-task walking in pwMS and HC. We expected that the fast 6MWT performed on a treadmill induces a deterioration in motor (spatio-temporal gait parameters) and/or cognitive performance (accuracy in calculating backward in steps of 3) associated with a change in PFC activation [relative oxy-/deoxyhemoglobin concentrations (HbO/HbR)] in pwMS, due to their limited attentional and/or cognitive capacity, but not in HC.

## Methods

### Participants

In total, 20 pwMS and 24 HC with similar age and sex were enrolled in this cross-sectional study. No sample size calculation was performed because comparable studies were lacking to obtain an effective size. However, our sample size was higher than those of other functional near-infrared spectroscopy (fNIRS) walking studies in pwMS (19, 22, 23). For inclusion, a MS diagnosis according to the revised McDonald criteria (24) had to be confirmed and the last acute episode as well as cortisone intake had to be at least 1 month ago. Furthermore, the expanded disability status scale (EDSS) (25) should not be higher than 4.5. This ensured that the subjects were able to walk at least 300 m at a stretch without aids. HC were excluded if any orthopedic, neurological, or untreated cardiovascular disease were present. The study procedure was approved by the ethics committee of the Medical Faculty of the Otto von Guericke University Magdeburg (Germany) (No.: 116/18).

### Study Procedure

**Figure 1:**
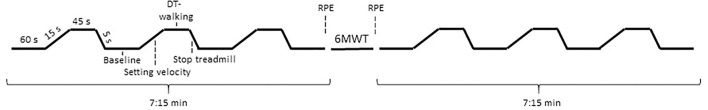
Experimental protocol. DT-walking, dual-task walking; 6MWT, 6-min walk test; RPE, rating of perceived exhaustion.

This study was conducted at the Otto von Guericke University Magdeburg (Germany) and the Kliniken Schmieder Konstanz (Germany). Patients with MS were recruited at the clinic by medical professionals during their admission to the rehabilitation clinic. The healthy subjects were recruited via a local newspaper article. The participants were informed about the study in a personal conversation and written informed consent was obtained. In total, the participants had three appointments: (i) pre-assessment of clinically relevant outcomes, (ii) familiarization, and (iii) the main measurement. All the measurements were done in the morning in a rested state with at least 24 h between sessions. During the pre-assessment, questionnaires were filled in [12-Item Multiple Sclerosis Walking Scale (MSWS-12) (26), Fatigue Scale for Motor and Cognitive function (FSMC) (27) and Beck Depression Inventory-II (BDI-II) (28)] and the 6MWT (29) was performed. Before and after the 6MWT, subjects were asked to rate their perceived exhaustion (RPE) using a Borg scale (6 = no exhaustion, 20 = maximal exhaustion). In the familiarization session, the comfort walking velocity on the treadmill was determined, the test protocol was explained in detail, the measurement equipment was applied and the first block of the measurement protocol was carried out (see Figure 1). A block design recommended for fNIRS recordings was used (30) during which the subjects had to alternate between standing (baseline) and dual-task walking every 60 s. Throughout the standing phase (baseline, 60 s), the participants should stand as stable as possible with hands on the rails without talking. Afterwards, the treadmill was accelerated to the prior determined individual comfort velocity within 15 s. During the subsequent dual-task walking (45 s), the subject had to calculate backwards by 3 from a randomly chosen number between 300 and 400 as it was used previously by Mofateh et al. (31). The subjects were told beforehand that if they make a mistake they should continue with the calculations and they were not corrected by the instructor. If they continued to calculate correctly backwards by 3 after the error, the answers were considered as correct. Finally, the treadmill was stopped within 5 s and the subjects stood still for further 60 s. This protocol was repeated three times in a row with a total duration of 7:15 min. The start and stop of the treadmill were announced loudly by the instructor.

The main measurements were conducted according to the above described dual-task-walking protocol prior and after the fast 6MWT performed on the same treadmill with comfort velocity plus 15% (see Figure 1 for more detail). Directly after the last standing phase of the pre-block, the treadmill was started and the subjects should concentrate on walking only for 6 min. Subsequently, the participants started with the first baseline measurement (standing) of the post-block. Before and after the fast 6MWT, RPE was inquired as an index of perceived fatigability. The study was performed on the treadmill to protect the pwMS from falling, due to motor or cognitive exhaustion, by using a harness during walking.

### Equipment and Outcome Measures

The gait parameters were derived from the acceleration and gyroscope data acquired with inertial measurement units (IMUs/MTw, Xsens Technologies BV, The Netherlands) fixed dorsally at both feet. Data were recorded during the dual-task assessments and the fast 6MWT on the treadmill with a sampling frequency of 120 Hz. The spatio-temporal gait parameters were calculated based on the algorithms of Hamacher et al. (32). The outcome parameters were stride length, stride time, stance time, swing time, and the minimum toe clearance (MTC) as well as their relative variability expressed by the coefficient of variation [CV (%) = standard deviation/mean × 100]. If these parameters changed significantly, we have interpreted this as motor performance fatigability. Cognitive performance fatigability was evaluated by the change in accuracy rate (number of correct calculations and total errors) during dual-task walking from before to after the fast 6MWT.

Two portable continuous-wave fNIRS systems were utilized (NIRSport, NIRx Medical Technologies, New York, USA) each attached to a standardized cap with 56 and 58 cm circumference, respectively (EasyCap GmbH, Herrsching, Germany). The smaller cap was used for people with a head circumference of <57 cm and the larger one for ≥ 57 cm. Each fNIRS system is composed of eight sources and eight detectors as well as eight short-separation channels with an average source-detector distance of 30–40 mm. The wavelengths inherent to the system are 760 and 850 nm and the sampling frequency is fixed at 7.81 Hz. The placement over the PFC was done with the fNIRS Optodes' Location Decider (fOLD) toolbox (33). The sensitivity of the channels was described in Broscheid et al. (34). The cap was positioned with Cz centrally [according to the international 10–20 system for electroencephalography (35)] between the nasion and inion and preauricular points on the left and right side. To reduce the influence of ambient light, an additional darkening cap was placed over the system.

The PFC was subdivided into the right, left and medial dorsolateral PFC Brodmann area 9 and 46 (r/lDLPFC9, r/lDLPFC46, mDLPFC9), the right, left, and medial frontopolar cortex Brodmann area 10 (r/l/mFPC10) and the right and left Broca Brodmann area 45 (r/lBroca45). These subareas were built by the following channels: 17,20 and 22 (lDLPFC9); 1,18 and 21 (rDLPFC9); 13 (lDLPFC46); 6 (rDLPFC46); 19 (mDLPFC9); 10,11,12 and 14 (lFPC10); 4,5,7 and 8 (rFPC10); 9 (mFPC10); 15 and 16 (lBroca45); 2 and 3 (rBroca45). The outcome parameters were the mean HbO and HbR concentrations in the respective subareas during the dual-task walking protocol performed prior and after the fast 6MWT.

In order to control physiological fNIRS signal confounders, a 3-channel electrocardiography system (SOMNOtouch™ NIBP, SOMNOmedics GmbH, Germany) was applied and heart rate as well as heart rate variability (HRV; specified by the time interval between two R-spikes/RR-interval) were determined.

### Functional Near-Infrared Spectroscopy Data Processing

To process and convert the fNIRS data, Homer3 (version 1.32.4) was used (36). First, non-existing values were replaced by spline interpolation (function hmrR_PreprocessIntensity_NAN). Afterwards, channels with a too weak or too strong signal as well as a too high standard deviation were excluded (function hmrR_PruneChannels: data range = 1 × 10^−2^ to 1 × 10^7^; signal-to-noise threshold = 2; source detector separation range: 0.0–45.0 mm). The preprocessed raw data were then converted to optical density data (function hmR_Intensity2OD) (36). Using the spline interpolation and a digital Savitzky–Golay filter motion artifacts were removed (function hmR_MotionCorrectSplineSG: *p* = 0.99; frame size = 15 s) (37). Furthermore, the 3rd order Butterworth bandpass filter was applied to diminish physiological artifacts (function hmrR_BandpassFilt: Bandpass_Filter_OpticalDensity) (30). Therefore, the high-pass filter was set to 0.01 Hz to minimize the proportion of oscillations associated with vascular endothelial function (30) and the low-pass filter to 0.09 Hz to primarily filter out Mayer waves (38). Subsequently, the optical density data were converted to concentration data by the Beer–Lambert Law adapting the differential path length factor to the age of each participant (39). Finally, the individual hemodynamic response function (HRF) was calculated with the ordinary least squared deconvolution method by utilizing a general linear model approach (function hmrR_GLM) (40). Within this approach, the HRF was based on a consecutive sequence of Gaussian functions (width of the Gaussian 0.5 and temporal spacing between consecutive Gaussians 0.5). The short separation regression was performed with the nearest short separation channel. The 3rd order polynomial drift baseline correction was applied.

Afterwards, the data were post-processed in MATLAB (version R2020b, The MathWorks, Natick, Massachusetts, USA). First, the acceleration phase of the treadmill (15 s) and the early phase of task onset (15 s) were cut out for each subject to avoid transient effects of movement initiation on the hemodynamic response (41, 42). Second, the last 5 s were cut out to minimize the impact of the expected ending of the walking trial (41, 43). Accordingly, data recorded in the time interval 30–55 s from treadmill start to stop were analyzed. The HbO and HbR concentration data of this time interval of each channel were then averaged for each subject. Finally, the channels were merged to the subareas of the PFC described above.

### Statistics

Statistical analysis was performed using IBM SPSS software (version 26, Chicago, USA). Normal distribution was checked with the Shapiro–Wilk test indicating that the majority of the data were normally distributed. Repeated measures ANOVA (rmANOVA) were carried out with the factors time (dual-task assessments prior and after the fast 6MWT as well as for each minute of the fast 6MWT) and group (pwMS and HC). It was assumed, as described in Blanca et al. (44), that the rmANOVA is robust to violation of the normal distribution. If the sphericity was not given, the Greenhouse–Geisser correction was applied. The effect size was determined using partial eta-squared (η_p_^2^) (small > 0.01, medium > 0.06, large > 0.14 effect) (45). In case of significant main or interaction effects, Bonferroni *post-hoc* tests were conducted. For the within-group comparison the effect size Cohen's d was determined (small > 0.2, medium > 0.5, large > 0.8 effect size) (45, 46). For the between-group comparison the bias-corrected Hedges' g was used (small > 0.2, medium > 0.5, and large > 0.8 effect size) (46). Statistical significance was accepted at *p* ≤ 0.05. Since patient groups are mostly very heterogeneous and a small *p*-value does not have to be equivalent to clinical relevance (46, 47), also non-significant results were interpreted, if they showed at least a medium effect size (η_p_^2^ > 0.06; *d* > 0.5; *g* > 0.5).

## Results

### Participants Characteristics and Clinical Outcomes

In total, 20 pwMS (13 females/7 males; 48.3 ± 9.0 years; 173.9 ± 9.1 cm; 75.7 ± 11.1 kg) and 24 HC (17 females/7 males; 48.6 ± 7.9 years; 171.7 ± 8.2 cm; 72.2 ± 12.6 kg) were included in the study. The pwMS were mildly to moderately affected (EDSS of 2.7 ± 1.0) and had an average disease duration of 14.0 ± 8.4 years since the first diagnosis. Sixteen pwMS were classified as the relapsing-remitting MS-type, two pwMS as the secondary, and two as the primary progressive MS-type. The pwMS reported moderate perceived walking limitations (MSWS-12: 53.8 ± 20.3%) and severe perceived trait fatigue (FSMC_total_: 68.1 ± 10.9; FSMC_cognitive_: 33.5 ± 10.1; FSMC_physical_: 34.5 ± 9.3). The BDI-II was higher in pwMS (11.3 ± 8.0) than HC (3.0 ± 3.3), but in both cases not conspicuous with regard to depression.

During the overground 6MWT (clinical pre-assessment), the pwMS covered a distance of 470.3 ± 71.3 m and the HC 639.0 ± 56.2 m. Based on the distance walked index (11, 48), four pwMS displayed motor performance fatigability during the 6MWT. However, if the second minute was taken as the baseline for the calculation of the distance walked index as it was recommended by Broscheid et al., it was only one person (13).

### Dual-Task Performance

#### Gait Performance

Gait data of three HC and one pwMS could not be analyzed due to poor data quality. The comfort velocity on the treadmill was 3.0 ± 0.7 km/h in pwMS and 4.8 ± 0.4 km/h in HC.

**Figure 2:**
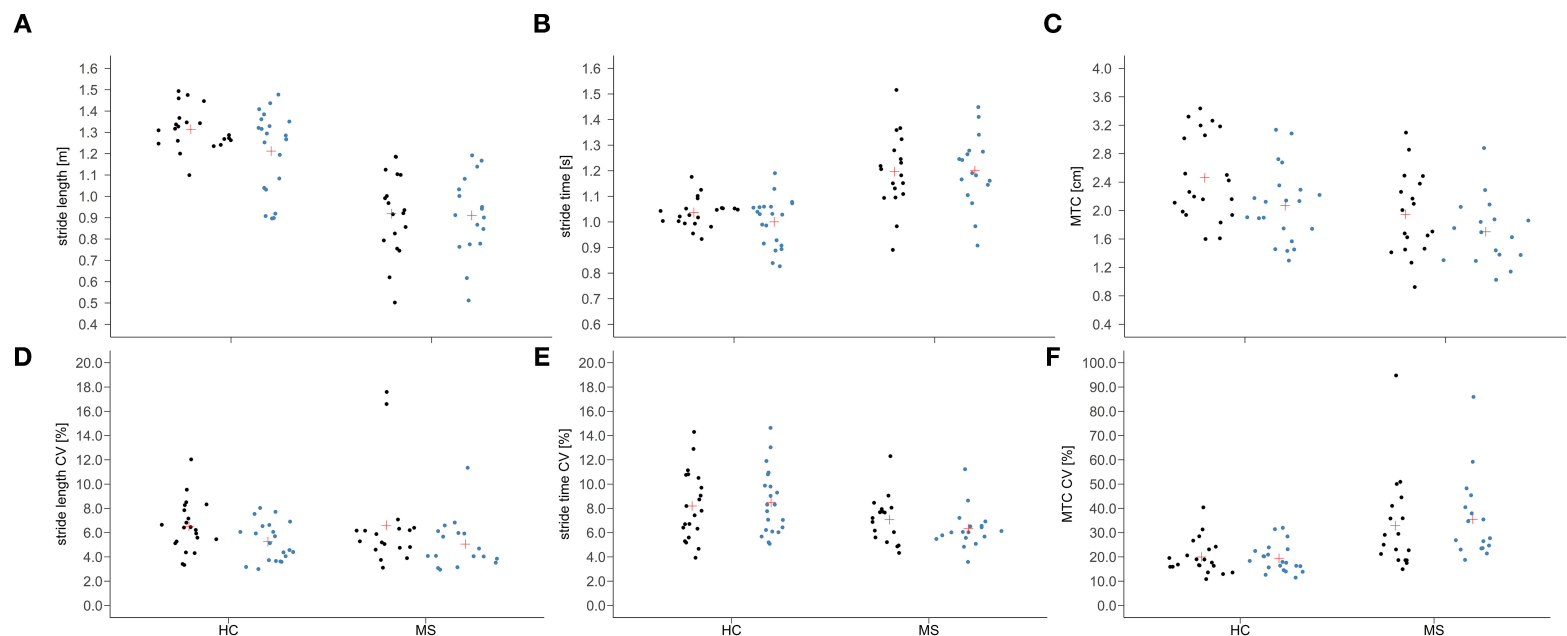
Individual data (dots) and means (red cross) of stride length **(A)**, stride time **(B)**, and minimum toe clearance [MTC; **(C)**] as well as their coefficient of variation [CV; **(D–F)**] before (black) and after (blue) the fast 6-min walk test in people with multiple sclerosis (MS) and healthy controls (HC).

Group × time interactions could be proven for MTC (*p* = 0.021; η_p_^2^ = 0.13), stride length (*p* = 0.019; η_p_^2^ = 0.14) and swing time (*p* = 0.033; η_p_^2^ = 0.11). A group × time interaction with medium effect size was shown for the MTC_CV_ (*p* = 0.124; η_p_^2^ = 0.06) and the stance time_CV_ (*p* = 0.119; η_p_^2^ = 0.06). The mean and individual data of these gait parameters and their CV are illustrated in Figure 2. Time effects for the gait parameters MTC (*p* < 0.001; η_p_^2^ = 0.52), stride length (*p* = 0.050; η_p_^2^ = 0.10) and stride length_CV_ (*p* < 0.001; η_p_^2^ = 0.30) were found (Table 1). For the stride time (*p* = 0.073; η_p_^2^ = 0.08) and the stance time (*p* = 0.107; η_p_^2^ = 0.07), the time effect was non-significant but a medium effect size was present. Group effects were shown for all the spatio-temporal gait parameters (*p* < 0.05; η_p_^2^ = 0.13–0.57) except stride length_CV_ and stride time_CV_.

**Table 1:** Spatio-temporal gait parameters recorded during dual-task walking before and after the fast 6MWT (mean ± standard deviation) and rmANOVA outcomes (*p*-values and effect size partial eta^2^).

**Gait parameter**	**Group**	**Dual-task walking performance pre/post 6MWT**		***p*** **-value**			**Partial eta** ^**2**^		
		**Pre**	**Post**	**T**	**G**	**GxT**	**T**	**G**	**GxT**
MTC [cm]	pwMS	1.96 ± 0.57	1.78 ± 0.51	**0.000**	**0.025**	**0.021**	**0.52**	**0.13**	**0.13**
	HC	2.46 ± 0.60	2.07 ± 0.52						
MTC_CV_ [%]	pwMS	32.50 ± 18.73^#^	35.00 ± 16.21^#^	0.319	**0.001**	0.124	0.03	**0.25**	0.06
	HC	19.97 ± 7.05^#^	19.43 ± 5.80						
Stride length [m]	pwMS	0.91 ± 0.19	0.92 ± 0.19	**0.050**	**0.000**	**0.019**	**0.10**	**0.57**	**0.14**
	HC	1.31 ± 0.10	1.21 ± 0.19^#^						
Stride length_CV_ [%]	pwMS	6.67 ± 3.86^#^	5.11 ± 1.96^#^	**0.000**	0.997	0.682	**0.30**	0.00	0.00
	HC	6.53 ± 2.09	5.26 ± 1.59						
Stance time [s]	pwMS	0.61 ± 0.09	0.61 ± 0.09	0.107	**0.013**	0.284	0.07	**0.15**	0.03
	HC	0.56 ± 0.04	0.55 ± 0.06						
Stance time_CV_ [%]	pwMS	16.69 ± 10.73^#^	17.34 ± 11.77^#^	0.555	**0.002**	0.119	0.01	**0.22**	0.06
	HC	9.68 ± 3.60^#^	8.27 ± 1.31						
Swing time [s]	pwMS	0.57 ± 0.19^#^	0.58 ± 0.18^#^	0.347	**0.010**	**0.033**	0.02	**0.16**	**0.11**
	HC	0.47 ± 0.02	0.45 ± 0.04						
Swing time_CV_ [%]	pwMS	28.17 ± 27.93^#^	19.12 ± 8.77	0.453	**0.013**	0.278	0.02	**0.15**	0.03
	HC	15.34 ± 6.68^#^	16.47 ± 7.87						
Stride time [s]	pwMS	1.18 ± 0.15	1.19 ± 0.14	0.146	**0.000**	0.073	0.06	**0.38**	0.08
	HC	1.04 ± 0.06	1.00 ± 0.10						
Stride time_CV_ [%]	pwMS	12.06 ± 21.72^#^	6.35 ± 1.57^#^	0.271	0.724	0.221	0.03	0.00	0.04
	HC	8.20 ± 2.80	8.51 ± 2.67						

*MTC, minimum toe clearance; CV, coefficient of variation; pwMS, people with multiple sclerosis; HC, healthy controls; 6MWT, 6-min walk test; T, time effect; G, group effect; GxT, group x time effect; bold, p-value ≤ 0.05*;

# *non-normally distributed*.

The *post-hoc* within group comparisons revealed that the stance time (*p* = 0.010; *d* = 0.1) decreased and the stride time (*p* < 0.001; *d* = 0.1) increased significantly after the fast 6MWT in pwMS (Supplementary Table 1). The MTC and stride length_CV_ decreased with a large (*p* = 0.087; *d* = 1.0) and a medium effect size (*p* = 0.793; *d* = 0.5), respectively, in pwMS. For the HC, it was shown that the MTC (*p* = 0.010; *d* = 1.1), stride length (*p* = 0.002; *d* = 0.5), stance time (*p* = 0.028; *d* = 0.4), swing time (*p* = 0.028; *d* = 0.5) and stride time (*p* < 0.001; *d* = 0.4) decreased significantly from pre to post of the fast 6MWT. The stride length_CV_ decreased with a large effect size (*p* = 0.883; *d* = 0.9).

The *post-hoc* between group comparisons indicated that both groups differed significantly in MTC (pre: *p* = 0.010; *g* = 0.8; post: *p* = 0.087; *g* = 0.5), stride length (pre: *p* < 0.001; *g* = 2.7; post: *p* < 0.001; *g* = 1.5), swing time (pre: *p* = 0.028; *g* = 0.7), MTC_CV_ (pre: *p* = 0.007; *g* = 0.88; post: *p* < 0.001; *g* = 1.3), and stance time_CV_ (pre: *p* = 0.007; *g* = 0.88; post: *p* = 0.001; *g* = 1.1). However, a medium effect size was proven for swing time (post: *p* = 0.087; *g* = 0.5).

#### Cognitive Performance

No significant group × time interaction, time or group effects were demonstrated for the total number of errors (pwMS pre: 0.3 ± 0.5/post: 0.7 ± 1.2; HC pre: 0.8 ± 1.0/post: 0.9 ± 0.9) and total number of correct calculations (pwMS pre: 18.0 ± 5.4/post: 18.4 ± 6.7; HC pre: 20.4 ± 8.3/post: 20.9 ± 9.5) during dual-task walking. However, for both, the total number of errors (*p* = 0.052; η_p_^2^ = 0.09) and correct calculations (*p* = 0.110; η_p_^2^ = 0.06), a time effect with a medium effect size was shown. The within group *post-hoc* tests indicated a significant increase in the error rate after the fast 6MWT in pwMS (*p* = 0.028; *d* = 0.6) but not in HC (*p* = 0.596; *d* = 0.1) (Supplementary Table 1).

#### Prefrontal Cortex Hemodynamics

Due to poor signal quality, the fNIRS data of two pwMS had to be excluded from the statistical analysis. No significant group × time interaction, time, or group effects were found for HbO and HbR for all PFC subareas (Table 2). A medium effect size was demonstrated for the group × time interaction for HbR in rFPC10 (*p* = 0.124; η_p_^2^ = 0.06). Moreover, a time effect with a medium effect size was observed for HbO in lBroca45 (*p* = 0.102; η_p_^2^ = 0.07) and for HbR in mFPC10 (*p* = 0.132; η_p_^2^ = 0.06) (Table 2).

**Table 2:** Oxy-/deoxyhemoglobin concentrations in the subareas of the prefrontal cortex recorded during dual-task walking before and after the fast 6MWT (mean ± standard deviation) and rmANOVA outcomes (*p*-values and effect size partial eta^2^).

		**Oxyhemoglobin concentration**								**Deoxyhemoglobin concentration**							
				***p*** **-values**			**Partial eta** ^**2**^					***p*** **-values**			**Partial eta** ^**2**^		
**Parameter**	**Group**	**Pre**	**Post**	**T**	**G**	**GxT**	**T**	**G**	**GxT**	**Pre**	**Post**	**T**	**G**	**GxT**	**T**	**G**	**GxT**
lDLPFC9 [μmol/l]	pwMS	0.291 ± 0.614	0.199 ± 0.531	0.216	0.416	0.625	0.04	0.02	0.01	−0.066 ± 0.191	−0.007 ± 0.249^#^	0.475	0.489	0.441	0.01	0.01	0.02
	HC	0.387 ± 0.515^#^	0.177 ± 0.695							−0.010 ± 0.178^#^	−0.012 ± 0.174						
rDLPFC9 [μmol/l]	pwMS	0.071 ± 0.652^#^	0.251 ± 0.261	0.600	**0.043**	0.261	0.01	**0.10**	0.03	−0.006 ± 0.169^#^	−0.063 ± 0.198	0.523	0.313	0.292	0.01	0.03	0.03
	HC	0.451 ± 0.499	0.385 ± 0.627^#^							−0.089 ± 0.203	−0.075 ± 0.224^#^						
lDLPFC46 [μmol/l]	pwMS	0.191 ± 0.487^#^	0.134 ± 0.669	0.683	0.781	0.914	0.00	0.00	0.00	−0.131 ± 0.356^#^	−0.166 ± 0.364	0.548	0.184	0.933	0.01	0.04	0.00
	HC	0.105 ± 0.813	0.007 ± 1.010							−0.019 ± 0.327	−0.065 ± 0.313						
rDLPFC46 [μmol/l]	pwMS	−0.025 ± 0.678	−0.022 ± 0.703	0.218	0.236	0.212	0.04	0.04	0.04	−0.071 ± 0.210	−0.175 ± 0.337	0.144	0.601	0.469	0.05	0.01	0.01
	HC	0.415 ± 0.811	0.020 ± 0.880							−0.126 ± 0.255	−0.162 ± 0.208						
mDLPFC9 [μmol/l]	pwMS	0.241 ± 0.529^#^	0.177 ± 0.611^#^	0.923	0.988	0.500	0.00	0.00	0.01	0.024 ± 0.154	0.047 ± 0.142	0.879	0.105	0.675	0.00	0.06	0.00
	HC	0.107 ± 0.587	0.192 ± 0.455							−0.031 ± 0.195^#^	−0.042 ± 0.244						
lFPC10 [μmol/l]	pwMS	0.327 ± 0.533^#^	0.041 ± 0.841^#^	0.443	0.239	0.343	0.02	0.03	0.02	−0.043 ± 0.198^#^	−0.082 ± 0.305^#^	0.692	0.584	0.238	0.00	0.01	0.04
	HC	0.119 ± 0.845	0.150 ± 0.486							−0.035 ± 0.283	0.042 ± 0.245						
rFPC10 [μmol/l]	pwMS	−0.170 ± 0.826	−0.070 ± 0.742^#^	0.777	**0.011**	0.697	0.00	**0.15**	0.00	0.148 ± 0.387^#^	0.001 ± 0.322	0.541	0.263	0.124	0.01	0.03	0.06
	HC	0.342 ± 0.561	0.326 ± 0.763^#^							−0.009 ± 0.248	0.056 ± 0.289						
mFPC10 [μmol/l]	pwMS	−0.141 ± 0.444	0.024 ± 0.689	0.486	0.058	0.598	0.01	0.09	0.01	−0.046 ± 0.639^#^	0.067 ± 0.508^#^	0.132	0.750	0.984	0.06	0.00	0.00
	HC	0.201 ± 0.641	0.224 ± 0.759							−0.024 ± 0.279	0.087 ± 0.233						
lBroca45 [μmol/l]	pwMS	0.234 ± 0.651^#^	0.019 ± 0.516	0.102	0.485	0.933	0.07	0.01	0.00	−0.161 ± 0.304	−0.185 ± 0.174	0.256	0.056	0.507	0.03	0.09	0.01
	HC	0.367 ± 0.609	0.130 ± 0.886^#^							−0.017 ± 0.229	−0.108 ± 0.267^#^						
rBroca45 [μmol/l]	pwMS	0.059 ± 0.587	0.103 ± 0.434	0.455	0.189	0.306	0.01	0.04	0.03	−0.115 ± 0.291	−0.159 ± 0.316	0.549	0.275	0.785	0.01	0.03	0.00
	HC	0.377 ± 0.692	0.095 ± 0.748							−0.049 ± 0.303	−0.066 ± 0.233^#^						

*l/r/mDLPFC9, left/right/medial dorsolateral prefrontal cortex Brodmann area 9; l/r/mDLPFC46, left/right dorsolateral prefrontal cortex Brodmann area 46; l/r/mFPC10, left/right/medial frontopolar cortex Brodmann area 10; l/rBroca45, left/right broca area Brodmann area 45; T, time effect; G, group effect; GxT, group x time effect; pwMS, people with Multiple Sclerosis; HC, healthy controls; bold, p-value ≤ 0.05*;

# *non-normally distributed*.

A significant group effect was detected for HbO in rDLPFC9 (*p* = 0.043; η_p_^2^ = 0.10) and in rFPC10 (*p* = 0.011; η_p_^2^ = 0.15). Moreover, a medium effect size for the group effect was shown for HbO in mFPC10 (*p* = 0.058; η_p_^2^ = 0.09) and for HbR in mDLPFC9 (*p* = 0.105; η_p_^2^ = 0.06) as well as in lBroca45 (*p* = 0.056; η_p_^2^ = 0.09).

The within group *post-hoc* tests did not reveal any significant differences (Supplementary Table 1). In the between groups *post-hoc* test, a higher HbR concentrations with a medium effect size were found for the rFPC10 (pre: *p* = 0.100; *g* = 0.5) in pwMS compared to HC.

**Figure 3:**
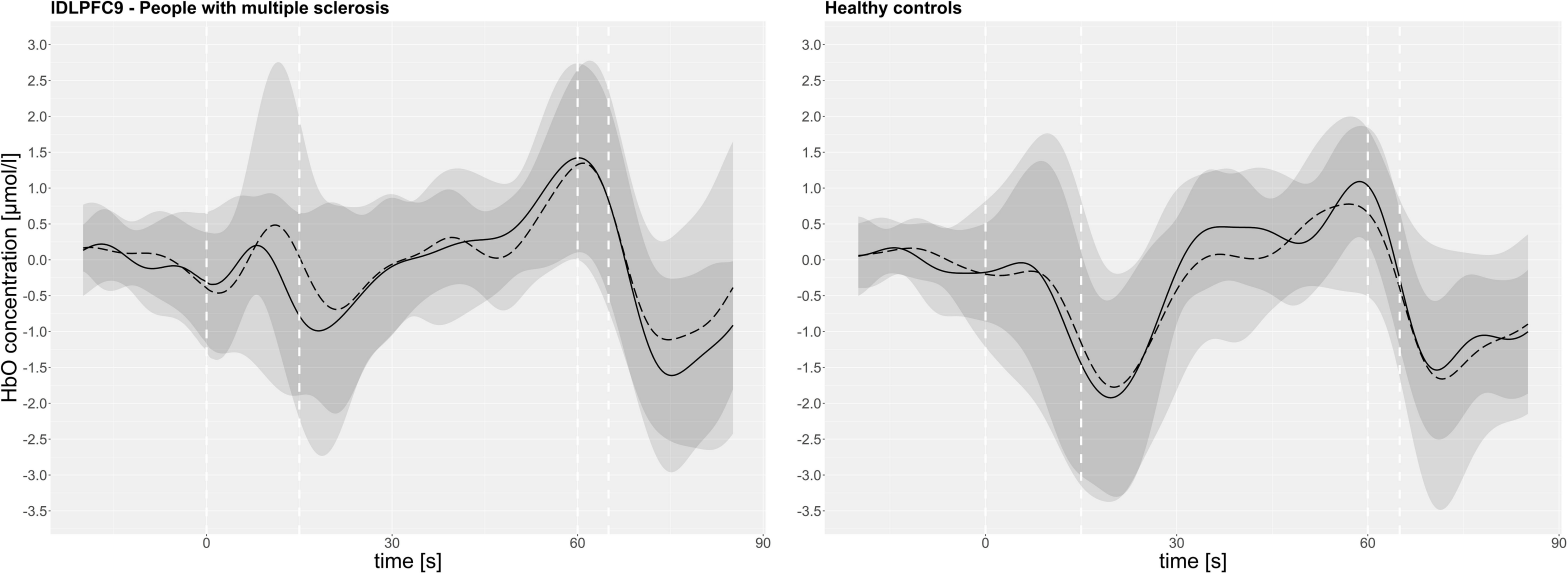
The oxyhemoglobin concentration (HbO) in the left dorsolateral prefrontal cortex Brodmann area 9 (lDLPFC9) of all subjects (mean pre: continuous line, post: dashed line/standard deviation pre: dark gray, post: light gray) over the course of measurement intervals. The dashed white vertical line represents: 0 s = start of the treadmill, 15 s = start of dual-task walking, 60 s = treadmill stopped, 65 s = start of the baseline.

The time course of HbO (before and after the fast 6MWT) averaged for pwMS and HC, respectively, is exemplarily displayed for the lDLPFC9 in Figure 3. The mean group data show that HbO increased after the start of the treadmill and dropped sharply in both groups, when the target velocity was reached. With a small time delay, after the start of the dual-task walking, the HbO concentration rose steadily above the initial level until the treadmill was stopped. Furthermore, the figure indicates that the standard deviation was particularly large during the acceleration of the treadmill (0–15 s) in both groups.

#### Heart Rate and Heart Rate Variability

Heart rate (pre 94.36 ± 10.82 bpm/post: 94.10 ± 9.41 bpm) and RR-interval (pre: 647.11 ± 73.88 ms/post: 646.22 ± 64.07 ms) remained stable from before to after the fast 6MWT in pwMS. In HC, the heart rate increased (pre: 94.38 ± 12.17 bpm/post: 98.00 ± 14.51 bpm) and the RR-interval decreased (pre: 660.87 ± 78.60 ms/post: 633.84 ± 87.40 ms). Along with this, group × time interaction and time effects were observed for heart rate (time: *p* = 0.028; η_p_^2^ = 0.13; time × group: *p* = 0.012; η_p_^2^ = 0.16) as well as the RR-interval (time: *p* = 0.004; η_p_^2^ = 0.21; time × group: *p* = 0.007; η_p_^2^ = 0.19). The within *post-hoc* tests revealed that the increase in heart rate (*p* = 0.001; *d* = 0.9) and the decrease in RR-interval (*p* < 0.001; *d* = 0.2) were significant in HC (Supplementary Table 1). The between-group *post-hoc* tests showed no significant differences nor medium effect sizes for the heart rate and RR-interval.

### 6-Min Walk Test

#### Gait Performance

For the gait parameters recorded during the fast 6MWT on the treadmill, group × time interaction and time effects were found for stride length_CV_ (time: *p* < 0.001; η_p_^2^ = 0.20; group × time: *p* < 0.001; η_p_^2^ = 0.24) and stride time_CV_ (time: *p* < 0.001; η_p_^2^ = 0.17; group × time: *p* < 0.001; η_p_^2^ = 0.22) (Table 3). Significant group effects were proven for all spatio-temporal gait parameters (*p* < 0.5; η_p_^2^ = 0.15–0.66) except for the MTC, which, however, exhibited a medium effect size (*p* = 0.079; η_p_^2^ = 0.08).

**Table 3:** Spatio-temporal gait parameters of every minute of the 6-min walk test (mean ± standard deviation) and rmANOVA outcomes (*p*-values and effect size partial eta^2^).

**Gait parameter**	**Group**	**Performance 6-min walk test**						***p*** **-values**			**Partial eta** ^**2**^		
		**1 min**	**2 min**	**3 min**	**4 min**	**5 min**	**6 min**	**T**	**G**	**GxT**	**T**	**G**	**GxT**
MTC [cm]	pwMS	2.23 ± 0.56	2.23 ± 0.54	2.23 ± 0.59	2.24 ± 0.60	2.28 ± 0.60	2.28 ± 0.62	0.538	0.079	0.222	0.02	0.08	0.04
	HC	2.65 ± 0.61	2.66 ± 0.63	2.59 ± 0.62	2.51 ± 0.59	2.49 ± 0.58	2.47 ± 0.59						
MTC_CV_ [%]	pwMS	30.90 ± 13.85^#^	30.83 ± 12.17	31.53 ± 16.02^#^	33.44 ± 14.94	30.45 ± 13.52^#^	31.83 ± 12.84^#^	0.496	**0.000**	0.500	0.02	**0.41**	0.02
	HC	17.90 ± 4.70	16.55 ± 4.79	16.61 ± 4.89	16.78 ± 5.56^#^	17.55 ± 5.35	20.96 ± 15.47^#^						
Stride length [m]	pwMS	1.06 ± 0.20	1.07 ± 0.20	1.07 ± 0.20	1.07 ± 0.20	1.08 ± 0.21	1.07 ± 0.23	0.511	**0.000**	0.808	0.01	**0.66**	0.00
	HC	1.45 ± 0.10	1.48 ± 0.11	1.48 ± 0.11	1.48 ± 0.11	1.48 ± 0.11	1.48 ± 0.11						
Stride length_CV_ [%]	pwMS	3.64 ± 2.26^#^	3.39 ± 2.16^#^	3.73 ± 2.44^#^	3.98 ± 3.04^#^	4.23 ± 2.75^#^	3.78 ± 1.63	**0.000**	**0.001**	**0.000**	**0.20**	**0.26**	**0.24**
	HC	4.56 ± 1.25	1.58 ± 0.43^#^	1.66 ± 0.40	1.67 ± 0.47	1.67 ± 0.46	1.93 ± 0.95^#^						
Stance time [s]	pwMS	0.62 ± 0.08	0.63 ± 0.08	0.63 ± 0.07^#^	0.63 ± 0.07	0.63 ± 0.08	0.63 ± 0.06	0.693	**0.000**	0.629	0.01	**0.51**	0.01
	HC	0.53 ± 0.03	0.52 ± 0.04	0.52 ± 0.04	0.53 ± 0.04	0.53 ± 0.04	0.53 ± 0.04						
Stance time_CV_ [%]	pwMS	8.75 ± 9.50^#^	9.11 ± 10.45^#^	9.51 ± 10.70^#^	9.55 ± 11.44^#^	9.12 ± 10.27^#^	9.18 ± 10.55^#^	0.309	**0.003**	0.174	0.03	**0.22**	0.05
	HC	6.17 ± 2.37	1.94 ± 0.60^#^	1.92 ± 0.59^#^	1.90 ± 0.52^#^	1.93 ± 0.61^#^	2.84 ± 3.47^#^						
Swing time [s]	pwMS	0.54 ± 0.18^#^	0.54 ± 0.18^#^	0.54 ± 0.17^#^	0.54 ± 0.17^#^	0.54 ± 0.17^#^	0.50 ± 0.11^#^	0.481	**0.015**	0.458	0.01	**0.15**	0.02
	HC	0.45 ± 0.02	0.45 ± 0.02	0.45 ± 0.02	0.45 ± 0.02	0.45 ± 0.02	0.45 ± 0.02						
Swing time_CV_ [%]	pwMS	9.78 ± 9.38^#^	9.27 ± 8.44^#^	10.09 ± 9.66^#^	10.06 ± 9.85^#^	10.11 ± 9.83^#^	11.65 ± 11.89^#^	0.302	**0.001**	0.528	0.03	**0.28**	0.01
	HC	4.61 ± 3.50^#^	2.29 ± 0.73	2.24 ± 0.77^#^	2.27 ± 0.76	2.26 ± 0.75	4.13 ± 7.81^#^						
Stride time [s]	pwMS	1.16 ± 0.14	1.17 ± 0.14	1.17 ± 0.13	1.17 ± 0.13	1.17 ± 0.13	1.13 ± 0.09	0.273	**0.000**	0.217	0.03	**0.53**	0.04
	HC	0.98 ± 0.05	0.97 ± 0.05	0.97 ± 0.05	0.98 ± 0.05	0.98 ± 0.06^#^	0.98 ± 0.06^#^						
Stride time_CV_ [%]	pwMS	2.90 ± 1.91^#^	2.68 ± 1.48^#^	3.09 ± 2.10^#^	3.24 ± 2.51^#^	3.97 ± 2.95^#^	3.55 ± 2.04^#^	**0.000**	**0.001**	**0.000**	**0.17**	**0.24**	**0.22**
	HC	4.37 ± 2.07	1.12 ± 0.33	1.07 ± 0.33	1.10 ± 0.32^#^	1.11 ± 0.39	1.88 ± 2.85^#^						

*MTC, minimum toe clearance; CV, coefficient of variation; pwMS, people with Multiple Sclerosis; HC, healthy controls; 6MWT, 6-min walk test; T, time effect; G, group effect; GxT, group x time effect; bold, p-value ≤ 0.05*;

# *non-normally distributed*.

The within-group *post-hoc* tests revealed that stride length_CV_ was lowest in the second minute and differed significantly from the third minute in pwMS (*p* = 0.13; *d* = 0.7) (Supplementary Table 2). Additionally, the second minute deviated with a medium effect size from the first (*p* = 1.000; *d* = 0.5) and fifth (*p* = 0.105; *d* = 0.5) minute in pwMS. In the latter, pwMS displayed the highest stride length_CV_. For the stride time_CV_, a non-significant difference but medium effect size was detected between the second and third (*p* = 1.000; *d* = 0.5) and fifth minute (*p* = 0.514; *d* = 0.5), respectively, in pwMS. Again, the stride time_CV_ was lowest in the second minute and highest in the fifth. In the HC, only the stride length_CV_ in the first minute was significantly higher than in the second (*p* < 0.001; *d* = 2.4). Nevertheless, a non-significant large effect size was shown for the difference between the second and the first minute in stride time_CV_ (*p* = 0.411; *d* = 1.6), which decreased from the first to the second minute.

The between-group *post-hoc* tests indicated that the groups differed in the minutes two to six of the fast 6MWT in both gait parameters stride length_CV_ (*p* < 0.01; *g* range = 1.2–1.4) and stride time_CV_ (*p* < 0.05; *g* range = 0.7–1.4) significantly. For the stride time_CV_, the groups differed also in the first minute (*p* = 0.027; *g* = 0.7).

#### Perceived Fatigability

No group × time interaction effect was observed. A significant time (*p* < 0.001; η_p_^2^ = 0.56) and group effect (*p* < 0.001; η_p_^2^ = 0.36) was demonstrated for the RPE (pwMS pre: 12.7 ± 2.6/post: 14.2 ± 2.5; HC pre: 9.5 ± 2.3/post: 10.6 ± 2.1).

The within group *post-hoc* tests revealed that the RPE was significantly increased after the fast 6MWT in both groups (pwMS: *p* < 0.001; *d* = 1.4; HC: *p* < 0.001; *d* = 0.8) (Supplementary Table 1).

## Discussion

This study investigated the effect of a fast 6MWT performed on a treadmill on motor and cognitive performance fatigability, perceived fatigability as well as PFC hemodynamics recorded during dual-task walking in pwMS and HC.

The main findings were that during the motor-cognitive dual-task (i) a distinct change in the spatio-temporal gait parameters toward a decreased MTC and stride length_CV_ and an increased stride time was observed in pwMS following the fast 6MWT. The HC displayed a change toward shorter and faster steps with less variability as well as a smaller MTC. Furthermore, (ii) cognitive performance during dual-task walking only declined in pwMS (increased error rate) and (iii) the PFC hemodynamics did not change in both groups. In addition, (iv) heart rate increased and HRV decreased only in HC after the fast 6MWT.

During the fast 6MWT, (v) stride length_CV_ and stride time_CV_ were lowest in the second minute and highest in the fifth minute in pwMS. In HC, both parameters were significantly higher in the first minute compared to the second. Lastly, (vi) both groups reported a slight but significant increase in perceived fatigue indicated by a higher RPE after the fast 6MWT.

There are only very few comparable studies investigating the impact of different fatiguing motor tasks on spatio-temporal gait parameters and the comparison should be made with caution as the fatigue protocols, the testing protocols (overground vs. treadmill walking), the task conditions (single vs. dual-task condition) and the calculation of the gait parameters (leg sides separated or averaged) were different. For instance, similar to our results, Granacher et al. reported a decreased stride length variability during dual-task overground walking in older adults after maximal isokinetic knee extensions (performed until they reached 50% of their maximal torque value) (49). Moreover, Nagano et al. investigated the influence of a fast 6MWT on spatio-temporal gait parameters [including the minimum foot clearance (MFC)] during 5 min treadmill single-task walking in young and older healthy adults (50). They found that the older adults exhibited a decreased MFC in the dominant leg, an increased MFC in the non-dominant leg and a decreased MFC variability in both legs after the fast 6MWT. In the present study, the MTC (averaged across both the legs) decreased slightly in both groups and the MTC_CV_ remained more or less stable.

In summary, our data imply that both pwMS and HC did not exhibit a clear indication of motor performance fatigability in this study. On the contrary, the observed changes in gait parameters might represent habituation to treadmill walking (51). In this regard, Meyer et al. investigated the change of kinematic gait parameters over 10 min single-task walking on a treadmill. They have found that toe height and step length variability decreased while stride time increased within the first 5 min. Thereafter these gait parameters remained stable (51). The same changes in these spatio-temporal gait parameters were observed in this study during dual-task walking. Thus, the fast 6MWT might have induced habituation to treadmill walking rather than motor performance fatigability. Nevertheless, these results have to be compared also with caution, because we have not observed this habituation effect during the fast 6MWT in both groups, but only during the dual-task walking afterward for most of these variables.

Regarding our initial hypothesis, we observed an indication for cognitive performance fatigability (increased error rate during the subtraction task), which might be due to the cognitive impairments (especially in the executive functions and attentional capacity) associated with MS (17).

This cognitive performance decline, without a clear sign of motor performance fatigability, might imply that the pwMS seemed to prioritize the motor over the cognitive task. Holtzer et al. described this with the posture first hypothesis during motor-cognitive dual-tasking (52). Nevertheless, Holtzer et al. also observed that the posture first hypothesis goes along with a higher PFC activation, which was not demonstrated in this study. One reason for these contrasting results could be that they performed overground single- and dual-task walking with a self-controlled walking velocity. In the present study, gait velocity of the participants was externally paced due to the treadmill. Several authors have shown that the PFC is primarily involved in the control of gait velocity and gait initiation (acceleration) (53–55). Thumm et al. compared the PFC activation during single-task overground and treadmill walking. They have demonstrated that the PFC activation was significantly lower during treadmill compared to overground walking in the Parkinson's disease (56). These findings differ from those of Herold et al., who compared, among other areas, the PFC activation during single-task overground and treadmill walking in healthy young adults (57). They demonstrated that the HbO concentrations in the left and right PFC were significantly higher during treadmill compared to overground walking. However, the age structure and health status differed between these as well as our study and are therefore only comparable to a limited extent.

Although the 6MWT was applied several times to investigate motor performance fatigability in pwMS (10), our data indicate that the duration and/or the intensity (comfort speed plus 15%) of the fast 6MWT was not sufficient to induce motor performance fatigability as well as changes in PFC activation at least in our sample of mildly to moderately affected pwMS. The heart rate and HRV data support this notion, which did not change in pwMS, but in HC. Especially the pwMS were rather cautious during the familiarization session when selecting the comfort walking velocity. Therefore, future studies should apply longer and/or more intense walking protocols to investigate motor performance and perceived fatigability in pwMS.

Another reason why motor performance fatigability was not clearly observed after the fast 6MWT might be related to the fNIRS block design. For the fNIRS baseline measurements, the subjects had to rest in a standing position for 60 s after the fast 6MWT and before the first dual-task walking interval was performed. Since the recovery of neural and muscular determinants of performance fatigability is fast after intense exercise, this break could have masked the real extent of the exercise-induced impairments (58, 59).

Finally, one limitation is that no single-task condition was performed to calculate dual-task costs. However, this was intentionally skipped because the exercise-induced impairments can disappear quickly (58, 59) and a single-task condition would have additionally increased the time delay between the fast 6MWT and the fatigue assessments. Moreover, the fNIRS cap can only be worn for a limited time due to the increasing pressure pain induced by the optodes at the forehead. Therefore, the measurements were kept as short as possible.

Another limitation is that some of the patients received disease modifying and symptomatic treatments, which may have had an impact on walking ability and with it on motor performance fatigability.

## Conclusion

In summary, cognitive performance fatigability during dual-task walking was demonstrated after the fast 6MWT on the treadmill in pwMS. However, no clear indication of motor performance fatigability was observed. Furthermore, heart rate and HRV remained stable in pwMS and both groups reported only a slight increase in ratings of perceived fatigue. Moreover, no change in the PFC activation was detected in both groups.

Future studies on this topic should increase the intensity and/or duration of the walking fatigue intervention to investigate its impact on performance and perceived fatigability measures in pwMS. Thereby, the level of disability should be considered. Additionally, further parameters such as muscular oxygenation (muscle NIRS) and/or myoelectrical activity (electromyography) should be recorded to detect if exercise intensity and/or duration was sufficient to induce alterations in neuromuscular function as done in studies investigation performance fatigability during single-joint exercise (60). Furthermore, the interactions of cognitive performance fatigability, motor performance fatigability, perceived fatigability as well as their neural correlates should not only be examined on treadmill, but also during overground walking that is closer to everyday walking. Altogether, this might help to detect fatigability in pwMS with the aim to improve therapeutic interventions.

## Data Availability Statement

The raw data supporting the conclusions of this article will be made available by the authors on request.

## Ethics Statement

The studies involving human participants were reviewed and approved by Ethics Committee of the Medical Faculty of the Otto von Guericke University Magdeburg/Leipziger Str. 44 / 39120 Magdeburg. The patients/participants provided their written informed consent to participate in this study.

## Author Contributions

K-CB, LS, and CD: conceptualization and methodology. K-CB: formal analysis and investigation. K-CB, MB, LS, and CD: interpretation of data. K-CB and MB: writing—original draft preparation. K-CB, MB, LS, CD, and MJ: writing—review and editing. LS, CD, and MJ: resources and supervision. All authors contributed to the article and approved the submitted version.

## Conflict of Interest

The authors declare that the research was conducted in the absence of any commercial or financial relationships that could be construed as a potential conflict of interest.

## Publisher's Note

All claims expressed in this article are solely those of the authors and do not necessarily represent those of their affiliated organizations, or those of the publisher, the editors and the reviewers. Any product that may be evaluated in this article, or claim that may be made by its manufacturer, is not guaranteed or endorsed by the publisher.

## References

[1] Braley TJ Chervin RD. Fatigue in multiple sclerosis: mechanisms, evaluation, and treatment. Sleep. (2010) 33:1061–7. 10.1093/sleep/33.8.1061 20815187 PMC2910465

[2] Guidelines, Multiple Sclerosis Clinical Practice. Fatigue and Multiple Sclerosis: Evidence-Based Management Strategies for Fatigue in Multiple Sclerosis. Washington, DC: Paralyzed Veterans of America (1998).

[3] Enoka RM Duchateau J. Translating fatigue to human performance. Med Sci Sports Exerc. (2016) 48:2228–38. 10.1249/MSS.0000000000000929 27015386 PMC5035715

[4] Kluger BM Krupp LB Enoka RM. Fatigue and fatigability in neurologic illnesses: proposal for a unified taxonomy. Neurology. (2013) 80:409–16. 10.1212/WNL.0b013e31827f07be 23339207 PMC3589241

[5] Enoka RM Duchateau J. Muscle fatigue: what, why and how it influences muscle function. J Physiol. (2008) 586:11–23. 10.1113/jphysiol.2007.139477 17702815 PMC2375565

[6] Tommasin S de Luca F Ferrante I Gurreri F Castelli L Ruggieri S. Cognitive fatigability is a quantifiable distinct phenomenon in multiple sclerosis. J Neuropsychol. (2020) 14:370–83. 10.1111/jnp.12197 31729168

[7] Behrens M Broscheid K-C Schega L. Taxonomie und determinanten motorischer performance fatigability bei multipler sklerose. NR. (2021) 27:3–12. 10.14624/NR2101001

[8] Behrens M Mau-Moeller A Lischke A Katlun F Gube M Zschorlich V. Mental fatigue increases gait variability during dual-task walking in old adults. J Gerontol A Biol Sci Med Sci. (2018) 73:792–7. 10.1093/gerona/glx210 29077783

[9] Venhorst A Micklewright D Noakes TD. Perceived fatigability: utility of a three-dimensional dynamical systems framework to better understand the psychophysiological regulation of goal-directed exercise behaviour. Sports Med. (2018) 48:2479–95. 10.1007/s40279-018-0986-1 30238409

[10] Severijns D Zijdewind I Dalgas U Lamers I Lismont C Feys P. The assessment of motor fatigability in persons with multiple sclerosis: a systematic review. Neurorehabilit Neural Repair. (2017) 31:413–31. 10.1177/1545968317690831 28413944

[11] Leone C Severijns D DoleŽalová V Baert I Dalgas U Romberg A. Prevalence of walking-related motor fatigue in persons with multiple sclerosis: decline in walking distance induced by the 6-minute walk test. Neurorehabilit Neural Repair. (2016) 30:373–83. 10.1177/1545968315597070 26216790

[12] Phan-Ba R Calay P Grodent P Delrue G Lommers E Delvaux V. Motor fatigue measurement by distance-induced slow down of walking speed in multiple sclerosis. PLoS ONE. (2012) 7:e34744. 10.1371/journal.pone.0034744 22514661 PMC3326046

[13] Broscheid K-C Behrens M Bilgin-Egner P Peters A Dettmers C Jöbges M. Instrumented assessment of motor performance fatigability during the 6-min walk test in mildly affected people with Multiple Sclerosis. Front. Neurol. 10.3389/fneur.2022.802516 PMC9125148 35614920

[14] Burschka JM Keune PM Menge U Oy UH Oschmann P Hoos O. An exploration of impaired walking dynamics and fatigue in Multiple Sclerosis. BMC Neurol. (2012) 12:161. 10.1186/1471-2377-12-161 23270547 PMC3547727

[15] Muir-Hunter SW Wittwer JE. Dual-task testing to predict falls in community-dwelling older adults: a systematic review. Physiotherapy. (2016) 102:29–40. 10.1016/j.physio.2015.04.011 26390824

[16] Leone C Patti F Feys P. Measuring the cost of cognitive-motor dual tasking during walking in multiple sclerosis. Mult Scler. (2015) 21:123–31. 10.1177/1352458514547408 25178543

[17] Rogers JM Panegyres PK. Cognitive impairment in multiple sclerosis: evidence-based analysis and recommendations. J Clin Neurosci. (2007) 14:919–27. 10.1016/j.jocn.2007.02.006 17659875

[18] Yogev G Hausdorff JM Giladi N. The role of executive function and attention in gait. Mov Disord. (2008) 23:329–472. 10.1002/mds.21720 18058946 PMC2535903

[19] Hernandez ME Holtzer R Chaparro G Jean K Balto JM Sandroff BM. Brain activation changes during locomotion in middle-aged to older adults with multiple sclerosis. J Neurol Sci. (2016) 370:277–83. 10.1016/j.jns.2016.10.002 27772776

[20] Covey TJ Zivadinov R Shucard JL Shucard DW. Information processing speed, neural efficiency, and working memory performance in multiple sclerosis: differential relationships with structural magnetic resonance imaging. J Clin Exp Neuropsychol. (2011) 33:1129–45. 10.1080/13803395.2011.614597 22047454

[21] Vuillerme N Forestier N Nougier V. Attentional demands and postural sway: the effect of the calf muscles fatigue. Med Sci Sports Exerc. (2002) 34:1907–12. 10.1097/00005768-200212000-00008 12471295

[22] Chaparro G Balto JM Sandroff BM Holtzer R Izzetoglu M Motl RW. Frontal brain activation changes due to dual-tasking under partial body weight support conditions in older adults with multiple sclerosis. J Neuroeng Rehabilit. (2017) 14:65. 10.1186/s12984-017-0280-8 28662727 PMC5493004

[23] Saleh S Sandroff BM Vitiello T Owoeye O Hoxha A Hake P. The role of premotor areas in dual tasking in healthy controls and persons with multiple sclerosis: an fNIRS imaging study. Front Behav Neurosci. (2018) 12:296. 10.3389/fnbeh.2018.00296 30618658 PMC6297844

[24] Polman CH Reingold SC Banwell B Clanet M Cohen JA Filippi M. Diagnostic criteria for multiple sclerosis: 2010 Revisions to the McDonald criteria. Ann Neurol. (2011) 69:292–302. 10.1002/ana.22366 21387374 PMC3084507

[25] Kurtzke JF. Rating neurologic impairment in multiple sclerosis: an expanded disability status scale (EDSS). Neurology. (1983) 33:1444–52. 10.1212/WNL.33.11.1444 6685237

[26] Hobart JC Riazi A Lamping DL Fitzpatrick R Thompson AJ. Measuring the impact of MS on walking ability: the 12-Item MS Walking Scale (MSWS-12). Neurology. (2003) 60:31–6. 10.1212/WNL.60.1.31 12525714

[27] Penner IK Raselli C Stöcklin M Opwis K Kappos L Calabrese P. The Fatigue Scale for Motor and Cognitive Functions (FSMC): validation of a new instrument to assess multiple sclerosis-related fatigue. Mult Scler. (2009) 15:1509–17. 10.1177/1352458509348519 19995840

[28] Hautzinger M Keller F Kühner C. BDI-II: Beck-Depressions-Inventar; Revision. Frankfurt am Main: Harcourt Test Services (2006).

[29] ATS Committee on Proficiency Standards for Clinical Pulmonary Function Laboratories. ATS Statement: guidelines for the six-minute walk test. Am J Respir Crit Care Med. (2002) 166:111–7. 10.1164/ajrccm.166.1.at1102 12091180

[30] Menant JC Maidan I Alcock L Al-Yahya E Cerasa A Clark DJ. A consensus guide to using functional near-infrared spectroscopy in posture and gait research. Gait Posture. (2020) 82:254–65. 10.1016/j.gaitpost.2020.09.012 32987345

[31] Mofateh R Salehi R Negahban H Mehravar M Tajali S. Effects of cognitive versus motor dual-task on spatiotemporal gait parameters in healthy controls and multiple sclerosis patients with and without fall history. Mult Scler Relat Disord. (2017) 18:8–14. 10.1016/j.msard.2017.09.002 29141826

[32] Hamacher D Hamacher D Taylor WR Singh NB Schega L. Towards clinical application: repetitive sensor position re-calibration for improved reliability of gait parameters. Gait Posture. (2014) 39:1146–8. 10.1016/j.gaitpost.2014.01.020 24602974

[33] Zimeo Morais GA Balardin JB Sato JR. fNIRS Optodes' Location Decider (fOLD): a toolbox for probe arrangement guided by brain regions-of-interest. Sci Rep. (2018) 8:3341. 10.1038/s41598-018-21716-z 29463928 PMC5820343

[34] Broscheid K-C Hamacher D Lamprecht J Sailer M Schega L. Inter-Session reliability of functional near-infrared spectroscopy at the prefrontal cortex while walking in multiple sclerosis. Brain Sci. (2020) 10:643. 10.3390/brainsci10090643 32957682 PMC7565127

[35] Salem Y Scott AH Karpatkin H Concert G Haller L Kaminsky E. Community-based group aquatic programme for individuals with multiple sclerosis: a pilot study. Disabil Rehabilit. (2011) 33:720–8. 10.3109/09638288.2010.507855 20726740

[36] Huppert TJ Diamond SG Franceschini MA Boas DA. HomER: a review of time-series analysis methods for near-infrared spectroscopy of the brain. Appl Opt. (2009) 48:D280–98. 10.1364/AO.48.00D280 19340120 PMC2761652

[37] Jahani S Setarehdan SK Boas DA Yücel MA. Motion artifact detection and correction in functional near-infrared spectroscopy: a new hybrid method based on spline interpolation method and Savitzky-Golay filtering. Neurophotonics. (2018) 5:15003. 10.1117/1.NPh.5.1.015003 29430471 PMC5803523

[38] Pinti P Scholkmann F Hamilton A Burgess P Tachtsidis I. Current status and issues regarding pre-processing of fNIRS neuroimaging data: an investigation of diverse signal filtering methods within a general linear model framework. Front Hum Neurosci. (2018) 12:505. 10.3389/fnhum.2018.00505 30687038 PMC6336925

[39] Scholkmann F Wolf M. General equation for the differential pathlength factor of the frontal human head depending on wavelength and age. J Biomed Opt. (2013) 18:105004. 10.1117/1.JBO.18.10.105004 24121731

[40] Ye JC Tak S Jang KE Jung J Jang J. NIRS-SPM: statistical parametric mapping for near-infrared spectroscopy. Neuroimage. (2009) 44:428–47. 10.1016/j.neuroimage.2008.08.036 18848897

[41] Lu CF Liu YC Yang YR Wu YT Wang RY. Maintaining gait performance by cortical activation during dual-task interference: A functional near-infrared spectroscopy study. PLoS ONE. (2015) 10:e0129390. 10.1371/journal.pone.0129390 26079605 PMC4469417

[42] Herold F Wiegel P Scholkmann F Müller NG. Applications of functional near-infrared spectroscopy (fNIRS) neuroimaging in exercise-cognition science: a systematic, methodology-focused review. J Clin Med. (2018) 7:466. 10.3390/jcm7120466 30469482 PMC6306799

[43] Nóbrega-Sousa P Gobbi LTB Orcioli-Silva D Conceição NRd Beretta VS Vitório R. Prefrontal cortex activity during walking: effects of aging and associations with gait and executive function. Neurorehabilit Neural Repair. (2020) 34:915–24. 10.1177/1545968320953824 32865134

[44] Blanca MJ Alarcón R Arnau J Bono R Bendayan R. Non-normal data: is ANOVA still a valid option? Psicothema. (2017) 29:552–7. 10.7334/psicothema2016.383 29048317

[45] Cohen J. Statistical Power Analysis for the Behavioral Sciences. 2nd ed. Hillsdale, NJ: Erlbaum (1988).

[46] Lakens D. Calculating and reporting effect sizes to facilitate cumulative science: a practical primer for t-tests and ANOVAs. Front Psychol. (2013) 4:863. 10.3389/fpsyg.2013.00863 24324449 PMC3840331

[47] Bhardwaj SS Camacho F Derrow A Fleischer AB Feldman SR. Statistical significance and clinical relevance: the importance of power in clinical trials in dermatology. Arch Dermatol. (2004) 140:1520–3. 10.1001/archderm.140.12.1520 15611433

[48] van Geel F Veldkamp R Severijns D Dalgas U Feys P. Day-to-day reliability, agreement and discriminative validity of measuring walking-related performance fatigability in persons with multiple sclerosis. Mult Scler. (2019) 26:1785–9. 10.1177/1352458519872465 31496362

[49] Granacher U Wolf I Wehrle A Bridenbaugh S Kressig RW. Effects of muscle fatigue on gait characteristics under single and dual-task conditions in young and older adults. J Neuroeng Rehabilit. (2010) 7:56. 10.1186/1743-0003-7-56 21062458 PMC2993724

[50] Nagano H James L Sparrow WA Begg RK. Effects of walking-induced fatigue on gait function and tripping risks in older adults. J Neuroeng Rehabilit. (2014) 11:155. 10.1186/1743-0003-11-155 25399324 PMC4253993

[51] Meyer C Killeen T Easthope CS Curt A Bolliger M Linnebank M. Familiarization with treadmill walking: how much is enough? Sci Rep. (2019) 9:5232. 10.1038/s41598-019-41721-0 30914746 PMC6435738

[52] Holtzer R Verghese J Allali G Izzetoglu M Wang C Mahoney JR. Neurological gait abnormalities moderate the functional brain signature of the posture first hypothesis. Brain Topogr. (2016) 29:334–43. 10.1007/s10548-015-0465-z 26613725 PMC4755880

[53] Harada T Miyai I Suzuki M Kubota K. Gait capacity affects cortical activation patterns related to speed control in the elderly. Exp Brain Res. (2009) 193:445–54. 10.1007/s00221-008-1643-y 19030850

[54] Mihara M Yagura H Hatakenaka M Hattori N Miyai I. [Clinical application of functional near-infrared spectroscopy in rehabilitation medicine]. Brain Nerve. (2010) 62:125–32. 10.11477/mf.1416100628 20192032

[55] Suzuki M Miyai I Ono T Oda I Konishi I Kochiyama T. Prefrontal and premotor cortices are involved in adapting walking and running speed on the treadmill: an optical imaging study. Neuroimage. (2004) 23:1020–6. 10.1016/j.neuroimage.2004.07.002 15528102

[56] Thumm PC Maidan I Brozgol M Shustak S Gazit E Shema Shiratzki S. Treadmill walking reduces pre-frontal activation in patients with Parkinson's disease. Gait Posture. (2018) 62:384–7. 10.1016/j.gaitpost.2018.03.041 29626840

[57] Herold F Aye N Hamacher D Schega L. Towards the neuromotor control processes of steady-state and speed-matched treadmill and overground walking. Brain Topogr. (2019) 32:472–6. 10.1007/s10548-019-00699-8 30680671

[58] Froyd C Millet GY Noakes TD. The development of peripheral fatigue and short-term recovery during self-paced high-intensity exercise. J Physiol. (2013) 591:1339–46. 10.1113/jphysiol.2012.245316 23230235 PMC3607875

[59] Husmann F Mittlmeier T Bruhn S Zschorlich V Behrens M. Impact of blood flow restriction exercise on muscle fatigue development and recovery. Med Sci Sports Exerc. (2018) 50:436–46. 10.1249/MSS.0000000000001475 29112627

[60] Behrens M Zschorlich V Mittlmeier T Bruhn S Husmann F. Ischemic preconditioning did not affect central and peripheral factors of performance fatigability after submaximal isometric exercise. Front Physiol. (2020) 11:371. 10.3389/fphys.2020.00371 32411014 PMC7199714

