# Supplementary material for: Effects of a 6-Min Treadmill Walking Test on Dual-Task Gait Performance and Prefrontal Hemodynamics in People With Multiple Sclerosis
Source: Front Neurol. 2022 Apr 7;13:822952. doi: 10.3389/fneur.2022.822952 (PMC9022001; doi:10.3389/fneur.2022.822952)
Supplement: Supplementary file 1 [file Table_1.docx]

Supplementary Material

Table 1. Within group post-hoc tests (p-value and effect size Cohens’ d)

| **Parameter** | **Group** | **Pre/post** | | **Group** | **Pre/post** | |
| --- | --- | --- | --- | --- | --- | --- |
|  |  | p-value | Cohens' d |  | p-value | Cohens' d |
| **MTC** | **pwMS** | 0.087 | 1.0 | **HC** | **0.010** | **1.1** |
| **MTC**_CV_ |  | 0.083 | 0.3 |  | 0.686 | 0.1 |
| **Stride length** |  | 0.776 | 0.3 |  | **0.002** | **0.5** |
| **Stride length**_CV_ |  | 0.793 | 0.5 |  | 0.883 | 0.9 |
| **Stance time** |  | **0.010** | **0.1** |  | **0.028** | **0.4** |
| **Stride time** |  | **0.000** | **0.1** |  | **0.000** | **0.4** |
| **Swing time** |  | 0.392 | 0.3 |  | **0.028** | **0.5** |
| **lBroca45 (HbO)** |  | 0.378 | 0.2 |  | 0.183 | 0.3 |
| **rFPC10 (HbR)** |  | 0.245 | 0.3 |  | 0.451 | 0.2 |
| **mFPC10 (HbR)** |  | 0.334 | 0.2 |  | 0.254 | 0.4 |
| **RPE** |  | **0.000** | **1.4** |  | **0.000** | **0.8** |
| **Heart rate** |  | 0.798 | 0.1 |  | **0.001** | **0.9** |
| **RR-intervall** |  | 0.889 | 0.0 |  | **0.000** | 0.2 |
| **Cog.-task error** |  | **0.028** | **0.6** |  | 0.596 | 0.1 |
| **Cog.-task correct** |  | 0.196 | 0.2 |  | 0.328 | 0.2 |

Abbreviations: MTC, minimum toe clearance; CV, coefficient of variation; lBroca45, left broca area Brodmann area 45; rFPC10, right frontopolar cortex Brodmann area 10; mFPC10, medial frontopolar cortex Brodmann area 10; HbO, oxyhemoglobin concentration; HbR, deoxyhemoglobin concentration; RPE, rating of perceived exhaustion; RR-interval, time interval between two R-spikes; Cog.-task error, total number of errors in the cognitive task; Cog.-task correct, total number correct calculations dual-task walking; pwMS, people with Multiple Sclerosis; HC, healthy controls; bold, p-value ≤ 0.05.
