# Supplementary material for: Effects of a 6-Min Treadmill Walking Test on Dual-Task Gait Performance and Prefrontal Hemodynamics in People With Multiple Sclerosis
Source: Front Neurol. 2022 Apr 7;13:822952. doi: 10.3389/fneur.2022.822952 (PMC9022001; doi:10.3389/fneur.2022.822952)
Supplement: Supplementary file 2 [file Table_2.docx]

Supplementary Material

Table 2. Within group Post-hoc tests of the second compared to the other minutes of the 6-min walk test (p-value and effect size Cohens’ d)

| **Parameter** | **Group** | **Min 1** | | **Min 3** | | **Min 4** | | **Min 5** | | **Min 6** | |
| --- | --- | --- | --- | --- | --- | --- | --- | --- | --- | --- | --- |
|  |  | p-value | Cohens' d | p-value | Cohens' d | p-value | Cohens' d | p-value | Cohens' d | p-value | Cohens' d |
| **Stride length**_CV_ | **pwMS** | 1.000 | 0.5 | **0.013** | **0.7** | 0.929 | 0.3 | 0.105 | 0.5 | 1.000 | 0.1 |
| **Stride time**_CV_ |  | 0.528 | 0.2 | 1.000 | 0.5 | 0.912 | 0.3 | 0.514 | 0.5 | 1.000 | 0.4 |
| **Stride length**_CV_ | **HC** | **0.000** | **2.4** | 1.000 | 0.3 | 1.000 | 0.4 | 1.000 | 0.3 | 1.000 | 0.4 |
| **Stride time**_CV_ |  | 0.411 | 1.6 | 1.000 | 0.3 | 0.642 | 0.1 | 1.000 | 0.0 | 1.000 | 0.3 |

Abbreviations: CV, coefficient of variation; Min, minute; pwMS, people with Multiple Sclerosis; HC, healthy controls; **bold, p-value ≤ 0.05**.
